# Envisioning the Future in Times of Global Uncertainty: Latent Profiles of Societal Threat Perceptions and Future-Oriented Resources Among University Students

**DOI:** 10.3390/ejihpe16080114

**Published:** 2026-08-07

**Authors:** Giorgio Maria Regnoli, Anna Parola, Andrea Zammitti, Barbara De Rosa

**Affiliations:** 1 Department of Humanities, University of Naples Federico II, Via Porta di Massa 1, 80133 Naples, Italy; giorgiomaria.regnoli@unina.it (G.M.R.); baderosa@unina.it (B.D.R.); 2 Department of Educational Sciences, University of Catania, 95124 Catania, Italy; andrea.zammitti@unict.it

**Keywords:** future anxiety, climate anxiety, fear of war, financial threat, polycrisis, latent profile analysis, future time perspective, university students

## Abstract

Young adults today are required to envision and plan for the future in a context marked by interconnected societal threats, such as climate change, geopolitical conflicts, and economic instability, giving rise to a condition of polycrisis. Understanding how these threats are perceived and their relationship with future-oriented psychological functioning is essential for promoting adaptive development during emerging adulthood. This study used a person-centered approach to identify different profiles of societal threat perception among university students and examine their links to social resources, future-oriented psychological resources, and well-being. The study involved a sample of 800 Italian university students. Latent profile analysis identified five distinct profiles. These profiles ranged from students who reported consistently low levels of perceived threat (Low Threat) to those characterized by elevated levels of threat across all domains (High Threat). Other profiles reflected specific concerns, such as heightened anxiety about climate change and war (Climate–War Concern), predominant financial threat concerns (Financially Focused Threat), and moderate threat perceptions combined with adaptive functioning (Moderate Threat). R3STEP analyses revealed that perceived social support and trust in institutions were significant predictors of profile membership. The lowest levels were observed among those with a High Threat profile. BCH analyses revealed significant differences in future orientation, meaning in life, and life satisfaction across profiles. Moderate Threat profile students reported the highest levels of future-oriented resources and well-being, whereas High Threat profile students reported the lowest. These findings suggest that university students differ in both the intensity of their societal threat perceptions and the configuration of these perceptions and their associated psychological resources. The results highlight the protective role of social and institutional resources, suggesting that adaptive functioning in times of global uncertainty depends less on the absence of concern and more on the capacity to maintain future-oriented psychological resources despite perceived threats.

## 1. Introduction

The transition to adulthood has always required young people to make important decisions about education, work, and personal relationships while imagining and planning their future (e.g., Arnett, 2000; Crocetti et al., 2012; Sica et al., 2024; Sestito & Sica, 2016; Schulenberg et al., 2004). Over the past decades, these developmental tasks have unfolded within a volatile, uncertain, complex, and ambiguous (VUCA) socioeconomic environment (Bennett & Lemoine, 2014), which has intensified emerging adults’ concerns regarding labor market entry, economic security, the attainment of autonomy, and future planning (Istituto Toniolo, 2024; EURES et al., 2024; Eurostat, 2026). This context can be understood through the lens of the risk society paradigm (Beck, 1992), according to which processes of modernization have generated global, systemic, and difficult-to-control risks that have transformed uncertainty into a structural feature of social life. Within this context, emerging adults are increasingly required to construct their own life trajectories, relying less on normative and predictable pathways while assuming greater personal responsibility for planning their futures.

The complexity of this context is further exacerbated by the growing exposure of emerging adults to global threats, including the climate crisis, geopolitical instability, armed conflicts, economic insecurity, and rapid social change. These threats do not operate independently; rather, they interact and reinforce one another, giving rise to a condition of polycrisis (Morin & Kern, 1993; Tooze, 2021, 2022) that heightens contextual unpredictability and makes it increasingly difficult to envision, plan, and pursue life projects. Within this framework, polycrisis represents not only a societal challenge but also a psychological one as it increases the burden of uncertainty that emerging adults are exposed to, with potential consequences for their mental health and their capacity to orient themselves toward the future (e.g., Barchielli et al., 2022; Carta et al., 2022; Romanello et al., 2024; UNICEF, 2023; Kałwak et al., 2024; World Health Organization, 2023).

For emerging adults, this context is particularly consequential because developmental tasks during this life stage are inherently future-oriented. Educational choices, career planning, financial independence, and family formation all rely on the assumption that the future is sufficiently stable to justify long-term investments. In this regard, the concept of futuring refers to the developmental capacity to imagine, anticipate, and actively construct future possibilities that provide direction for present decisions and identity development (Sica et al., 2016). When this capacity is constrained by a context characterized by instability and uncertainty, concerns about society are likely to become intertwined with concerns about one’s own future. The ability to maintain a positive vision of the future is therefore a key psychological resource for successfully navigating the developmental tasks of emerging adulthood, as a positive future orientation supports identity formation, goal setting, emotional regulation, and adaptive coping with uncertainty (e.g., Aspinwall & Leaf, 2002; Bellare et al., 2019; Fonseca et al., 2020; Negru, 2012; Regnoli et al., 2026a, 2026b; Shipp et al., 2009). However, increasing economic insecurity, difficulties in achieving autonomy, and the accumulation of global crises appear to foster increasingly negative, anxiety-laden, and catastrophic views of the future among young people (e.g., Bonanomi & Rosina, 2022; Mutia & Hargiana, 2021; Parrello, 2018; Regnoli et al., 2024a; Zaleski, 1996). Such representations may undermine their ability to envision meaningful and attainable future plans and to sustain positive expectations about their personal and professional futures. Understanding how young adults perceive and respond to contemporary societal threats has therefore become increasingly important for explaining differences in psychological adjustment and well-being.

What young adults experience, however, is unlikely to consist of a series of independent societal threats. Rather, they are simultaneously exposed to multiple and overlapping sources of uncertainty that may collectively shape how they imagine the future (e.g., Barchielli et al., 2022; Carta et al., 2022; Kałwak et al., 2024). From a polycrisis perspective (Tooze, 2021, 2022; Kałwak et al., 2024), contemporary uncertainty may be experienced across different yet interconnected levels, ranging from collective and macro-level risks to their more proximal socioeconomic consequences and, ultimately, to individuals’ expectations regarding their personal futures. Consistent with this multilevel framework, the four indicators used to identify the profiles were selected not as equivalent manifestations of a single source or dimension of distress, but as complementary expressions of the different ways in which contemporary societal instability, at both distal and proximal levels, may become embedded in young adults’ psychological experience. In the psychological literature, these different manifestations of contemporary uncertainty have been described through related yet conceptually distinct constructs, including climate anxiety (Clayton, 2020), fear of war (Kalcza-Janosi et al., 2023; Regnoli et al., 2023, 2024a), financial threat (Schoon & Mortimer, 2017), and future anxiety (Zaleski, 1996; Jannini et al., 2022).

Climate anxiety and fear of war primarily capture anticipatory emotional responses to large-scale collective threats, whereas financial threat reflects the perceived consequences of economic instability for individuals’ present and future material security. Future anxiety, by contrast, operates at a broader and more personal level, reflecting a generalized tendency to anticipate one’s future with apprehension and pessimism (Zaleski, 1996), through which multiple sources of uncertainty may converge into an overarching negative representation of what lies ahead. Despite differences in their level of analysis, domain specificity, and established terminology, these four constructs all capture anticipatory responses to conditions perceived as consequential, uncertain, and only partially controllable. Their joint examination was therefore intended to represent the multidimensional and multilevel nature of young adults’ experiences of polycrisis (e.g., Barchielli et al., 2022; Kałwak et al., 2024), while preserving the conceptual specificity of each research domain.

Within this framework, future anxiety is conceptually distinct from future time perspective. Whereas future anxiety reflects apprehension and negative expectations regarding one’s personal future, future time perspective constitutes a cognitive-motivational orientation that supports the formulation, planning, and pursuit of future goals (Kooij et al., 2018: Zimbardo & Boyd, 1999). Importantly, the two constructs should not be regarded merely as opposite poles of the same dimension. Experimental evidence suggests that an open future time perspective may coexist with negative affect while continuing to shape personal goal selection, indicating that anxiety about the future does not necessarily preclude future-oriented motivation and planning (Man & Turliuc, 2024). This interpretation is also consistent with research on climate anxiety, showing that emotional concern about large-scale threats may coexist with information seeking and collective engagement among young people (e.g., Regnoli et al., 2026a; Romano et al., 2024). Thus, individuals may experience apprehension about future conditions while retaining the capacity to formulate goals, invest in future possibilities, and engage in purposeful action. Future anxiety was therefore included as a profile indicator capturing a generalized negative orientation toward one’s personal future, whereas future time perspective was examined as a distinct psychological resource potentially supporting adaptive future-oriented functioning under conditions of uncertainty.

To date, most research has examined the impact of contemporary global challenges, including the constructs described above, using a predominantly unidimensional approach. Specifically, climate anxiety, fear of war, financial threat, and future anxiety have largely been investigated as distinct constructs, with evidence consistently linking each of them to poorer psychological adjustment, lower well-being, and greater emotional distress, particularly among young people (e.g., Clayton & Crandon, 2025; Chudzicka-Czupała et al., 2023; Cianconi et al., 2020; Hajek et al., 2023; Ogunbode et al., 2022; Regnoli et al., 2024b; Mottola et al., 2023; Massag et al., 2023; Bifulco & Dodaro, 2022; McWhirter & McWha-Hermann, 2021). Although these findings have contributed to clarifying the psychological implications of each individual crisis, they provide limited insight into how these different forms of threat, reflecting the complexity of contemporary global crises, coexist and are organized within individuals. This fragmented perspective may therefore fail to capture the cumulative and multidimensional nature of these societal concerns, as well as their potential joint influence on psychological functioning. Consequently, there is a need for a more integrated approach that simultaneously examines these constructs and identifies potential patterns of co-occurrence that may characterize distinct profiles of psychological vulnerability.

A person-centered perspective offers a useful way to address this limitation. Rather than focusing on isolated associations among variables, person-centered approaches identify subgroups characterized by distinct configurations of psychological characteristics. This perspective recognizes that individuals reporting similar levels of overall distress may nevertheless differ substantially in the specific societal threats they perceive as most salient. Understanding these configurations may therefore provide a more realistic account of how young adults experience uncertainty in contemporary society (e.g., Carta et al., 2022; Morin & Kern, 1993; Parola et al., 2026; Tooze, 2021, 2022; Valbusa et al., 2026; World Health Organization, 2023; Zammitti et al., 2026).

Most person-centered evidence currently comes from research on climate anxiety. Latent profile analyses have consistently shown that climate-related concerns are organized into qualitatively different patterns associated with distinct levels of psychological functioning and environmental engagement (Chen et al., 2026; Ferrajão et al., 2026; Karademas et al., 2026). Across studies, a consistent finding is that climate concern is not uniformly maladaptive. Individuals reporting moderate levels of concern often show greater psychological adjustment, stronger future orientation, and higher environmental engagement than both disengaged individuals and those experiencing pervasive distress (e.g., Bouman et al., 2020; Ogunbode et al., 2022; Innocenti et al., 2023). Conversely, profiles characterized by generalized concern and elevated distress consistently report poorer well-being and fewer psychological resources (e.g., Wullenkord et al., 2024; McBride et al., 2021; Schwaab et al., 2022).

Comparable evidence remains scarce for other societal threats. Existing person-centered studies on war-related concerns have mainly involved populations directly exposed to armed conflict, identifying distinct patterns of vulnerability and resilience (Olinover & Hamama, 2025; Amiri et al., 2026). By contrast, the psychological impact of war on individuals living in countries not directly involved in armed conflicts remains a relatively underexplored and insufficiently developed area within the psychological literature (e.g., Chudzicka-Czupała et al., 2023; Hajek et al., 2023; Regnoli et al., 2025). Studies conducted in the general population have predominantly adopted variable-centered approaches (Hajek et al., 2023; Regnoli et al., 2024a), focusing on average associations between fear of war and mental health outcomes, while providing limited insight into how war-related concerns coexist and interact with other forms of societal uncertainty at the individual level. Likewise, although financial threat has repeatedly been linked to anxiety, psychological distress, and lower subjective well-being (Marjanovic et al., 2013), little is known about whether economic insecurity combines with climate anxiety, fear of war, and future anxiety to form distinct psychological profiles. Consequently, current evidence provides only a partial understanding of how young adults organize multiple societal threats and whether different configurations are associated with different patterns of psychological adaptation.

A related question concerns why individuals reporting similar societal concerns differ in their psychological functioning. Adaptation to societal uncertainty is unlikely to depend exclusively on the intensity of perceived threats; it may also reflect the availability of psychological and contextual resources. Perceived social support and trust in institutions may reduce the impact of uncertainty by strengthening confidence in both interpersonal relationships and collective responses to societal challenges. In addition, subjective well-being represents an important indicator of psychological adaptation during emerging adulthood, with life satisfaction being widely considered one of its core cognitive components (e.g., E. D. Diener et al., 1985; E. Diener et al., 2018). Likewise, future-oriented resources, including future time perspective, meaning in life, and life satisfaction—may help explain why some young adults maintain positive expectations despite living in an uncertain social context (Parola et al., 2026; Sica et al., 2024). Examining these resources alongside patterns of societal threat may therefore provide a more comprehensive understanding of adaptation during emerging adulthood.

Against this background, the present study adopted a person-centered approach to investigate how Italian university students organize perceptions of climate anxiety, fear of war, financial threat, and future anxiety. Latent profile analysis was used to identify distinct configurations of societal threat. Subsequently, perceived social support and trust in institutions were examined as antecedents of profile membership whereas future time perspective, meaning in life, and life satisfaction were compared across profiles.

Given the exploratory nature of latent profile analysis, no specific hypotheses were formulated regarding the number or characteristics of the profiles. Nevertheless, we expected that profiles characterized by lower or more balanced perceptions of societal threats would report greater social and institutional resources, stronger future-oriented psychological functioning, and higher life satisfaction than profiles characterized by generalized perceptions of societal threat.

## 2. Materials and Methods

### 2.1. Participants and Procedure

The sample included 800 Italian university students aged between 18 and 34 years (M = 22.63, SD = 2.93). Of the participants, 60.5% identified as female (*n* = 484), 34.0% as male (*n* = 272), 4.5% as non-binary or another gender identity (*n* = 36), and 1.0% preferred not to disclose their gender (*n* = 8). Participants were enrolled across different years of study and represented a range of academic disciplines, including Psychology (26.6%), Engineering, Mathematics, and Computer Science (18.4%), Health (16.8%), Economics and Law (15.4%), and Humanities and Social Sciences (22.8%). Participants were recruited through convenience sampling and completed an online questionnaire. Faculty members were contacted and invited to disseminate the study among their students during lectures and through institutional communication channels. Students who voluntarily agreed to participate accessed the online questionnaire after providing informed consent. No incentives were provided. To be eligible for participation, individuals had to (a) be enrolled in an Italian university, (b) be between 18 and 34 years of age, and (c) provide informed consent. Responses were excluded if participants were younger than 18 years, were not currently enrolled in a university program, did not provide informed consent, or provided incomplete data for the study measures.

Participation was voluntary and anonymous, and no incentives were provided. Before completing the survey, participants were informed about the aims of the study, the confidentiality of their responses, and their right to withdraw from the study at any time without penalty. All participants provided informed consent prior to participation.

The study complied with the ethical guidelines of the Italian Association of Psychology (AIP) and was approved by the Ethics Committee of the University of Catania (Approval No.: Edunict-2025.04.24/03).

### 2.2. Measures

In addition to the collection of sociodemographic information (age, gender, year of study, and academic discipline), several psychological constructs were assessed using measures selected in accordance with the objectives of the study. Specifically, the following dimensions were examined:

***Future Anxiety.*** Future anxiety was assessed using the Dark Future Scale (DFS; Zaleski et al., 2019), specifically, the Italian version validated by Jannini et al. (2022). The scale consists of five items rated on a 7-point Likert scale ranging from 1 (=*decidedly false*) to 6 (=*decidedly true*). An example item is: “I am afraid that the problems which trouble me now will continue for a long time.” Higher scores indicate greater future anxiety. In the present study, Cronbach’s α was 0.90.

***Climate Anxiety.*** Climate anxiety was measured using the Climate Change Anxiety Scale (CCAS; Clayton & Karazsia, 2020), specifically, the Italian version validated by Innocenti et al. (2021). The scale assesses emotional and functional responses associated with concerns about climate change. The instrument includes 13 items covering cognitive-emotional impairment and functional impairment related to climate change concerns. Participants responded on a 5-point Likert scale ranging from 1 (=*never*) to 5 (=*almost always*). An example item is: “Thinking about climate change makes it difficult for me to concentrate”. Higher scores indicate greater climate anxiety. In the present study, Cronbach’s α was 0.93.

***Fear of war*.** Fear of war was measured using the Fear of War Scale (FOWARS; Kalcza-Janosi et al., 2023), specifically, the Italian version validated by Regnoli et al. (2023). The scale assesses emotional, cognitive, and physiological reactions associated with concerns about the possibility and consequences of armed conflict. The instrument includes 12 items covering two dimensions: Physiological Fear and Experiential Fear. Participants responded on a 5-point Likert scale ranging from 1 (=*strongly disagree*) to 5 (=*strongly agree*). An example item is: “The possibility of war worries me about my future”. Higher scores indicate greater war-related worry. In the present study, Cronbach’s α was 0.89.

***Financial Threat.*** Financial threat was measured using the Financial Threat Scale (FTS; Marjanovic et al., 2013). The scale assesses feelings of uncertainty, vulnerability, and perceived threat regarding one’s current and future financial situation. The instrument consists of five items rated on a 5-point Likert scale ranging from 1 (=*not at all*) to 5 (=*a great deal*). An example item is: “How threatened do you feel by your current financial situation?” Higher scores indicate greater perceived financial threat. Because the Financial Threat Scale has not been previously validated in Italian university samples, their factorial validity was examined through confirmatory factor analyses (CFAs). A one-factor model demonstrated excellent fit to the data (χ^2^_(5)_ = 6.94, *p* = 0.225, CFI = 0.999, TLI = 0.999, RMSEA = 0.022, 90% CI [0.000, 0.057], and SRMR = 0.006). All standardized factor loadings were statistically significant (*p* < 0.001). Cronbach’s α was 0.93.

***Future Time Perspective*.** Future time perspective was assessed using the Italian version of the Future Time Orientation Scale (FTOS; Coscioni et al., 2024), which was validated by (Santilli et al., 2025). The FTOS is an 8-item measure assessing the extent to which individuals’ current thoughts and behaviors are influenced by their psychological future. Participants responded using a Likert-type scale ranging from 1 (=*strongly disagree*) to 5 (=*strongly agree*). An example item is: “I value activities that may benefit me in the long run”. Higher scores indicate a stronger future time orientation. In the present study, Cronbach’s α was 0.89.

***Meaning in Life.*** Meaning in life was assessed using the Presence subscale of the Meaning in Life Questionnaire (MLQ; Steger et al., 2006), specifically, the Italian version validated by Negri et al. (2020). The measure consists of five items evaluating the extent to which individuals perceive their lives as meaningful and purposeful. Responses are provided on a 7-point Likert scale ranging from 1 (=*absolutely untrue*) to 7 (=*absolutely true*). An example item is: “My life has a clear sense of purpose.” Higher scores indicate greater perceived meaning in life. In the present study, Cronbach’s α was 0.84.

***Perceived Social Support.*** Perceived social support was measured using the Italian version of the Multidimensional Scale of Perceived Social Support (MSPSS; Zimet et al., 1988), which was validated by Busoni and Di Fabio (2008). The scale consists of 12 items assessing perceived support from family, friends, and significant others. Responses are provided on a 7-point Likert scale ranging from 1 (=*very strongly disagree*) to 7 (=*very strongly agree*). An example item is: “There is a special person who is around when I am in need.” Higher scores indicate greater perceived social support. In the present study, Cronbach’s α was 0.87 and McDonald’s ω was 0.87.

***Trust in Institutions.*** Trust in institutions was assessed through a brief 5-item measure developed for the purposes of the present study. The scale evaluates individuals’ confidence in the ability of public and international institutions to effectively address major contemporary societal challenges, including economic instability, climate change, and geopolitical conflicts. Participants responded on a 5-point Likert scale ranging from 1 (=*strongly disagree*) to 5 (=*strongly agree*). An example item is: “I trust public institutions to effectively address the major challenges facing society.” Higher scores indicate greater trust in institutions. Because the Trust in Institutions measure was developed for the purposes of the present study, its factorial validity was examined through a confirmatory factor analysis (CFA). A one-factor model was specified with the five items loading on a single latent construct. Results indicated an excellent fit to the data (χ^2^_(5)_ = 7.43, *p* = 0.191, CFI = 0.998, TLI = 0.996, RMSEA = 0.025, 90% CI [0.000, 0.059], and SRMR = 0.012). All standardized factor loadings were statistically significant (*p* < 0.001) and ranged from 0.65 to 0.68, supporting the unidimensional structure of the scale. Cronbach’s α was 0.80.

***Life Satisfaction.*** Life satisfaction was assessed using the Satisfaction with Life Scale (SWLS; E. D. Diener et al., 1985), specifically, the Italian version validated by Di Fabio and Busoni (2009). The SWLS consists of five items measuring the cognitive evaluation of one’s overall quality of life. Responses are provided on a 7-point Likert scale ranging from 1 (=*strongly disagree*) to 7 (=*strongly agree*). An example item is: “In most ways my life is close to my ideal”. Higher scores indicate greater life satisfaction. In the present study, Cronbach’s α was 0.89.

### 2.3. Data Analysis

Preliminary analyses, as descriptive statistics (means, standard deviations, skewness and kurtosis) and bivariate correlations among all study variables were conducted. Then, a confirmatory factor analysis (CFA) was performed. Subsequently, to identify distinct profiles of university students based on their perceptions of contemporary societal threats, a Latent Profile Analysis (LPA) was performed using four indicators: future anxiety, climate anxiety, fear of war, and financial threat. Latent profile models were estimated in Mplus Version 8.11 using the robust maximum likelihood estimator (MLR). To reduce the risk of converging on local maxima, models were estimated using 1000 random sets of starting values with 250 final-stage optimizations (STARTS = 1000 250) and 20 initial-stage iterations (STITERATIONS = 20). The best log-likelihood value was successfully replicated for the retained solution, indicating that the global maximum had been reached. Cases with missing values on the study variables were excluded prior to the analyses. Participants with missing data for the study variables were excluded prior to the analyses; therefore, the latent profile analyses were conducted using a complete-case dataset. Following a stepwise model selection strategy, increasingly complex models were estimated until the addition of further profiles no longer resulted in satisfactory improvements in model fit and/or produced poorly interpretable or unstable solutions. The optimal profile solution was identified based on multiple criteria. Specifically, the Akaike Information Criterion (AIC; Akaike, 1987), the Bayesian Information Criterion (BIC; Schwarz, 1978), and the sample-size adjusted Bayesian Information Criterion (SABIC; Sclove, 1987) were examined, with lower values indicating better model fit. Classification accuracy was evaluated using entropy, with values approaching 1 reflecting greater precision in profile assignment (Nagin, 2005). Model comparison was further supported by the Lo–Mendell–Rubin adjusted likelihood ratio test (LMR-LRT; Lo et al., 2001) and the Bootstrapped Likelihood Ratio Test (BLRT; McLachlan, 2000). These tests compare a model with k profiles to a model with k − 1 profiles, with significant *p*-values indicating that the model with k profiles provides a significantly better fit to the data. In addition to statistical fit indices, profile interpretability, theoretical meaningfulness, and profile size were considered when selecting the final solution. Finally, solutions containing profiles representing less than 5% of the sample were considered potentially unstable and interpreted with caution (Masyn, 2013).

Following the identification of the optimal profile solution, perceived social support and trust in institutions were included as antecedent variables using the R3STEP procedure (Asparouhov & Muthén, 2014a) to examine whether social resources were associated with latent profile membership. This approach estimates multinomial logistic regression models predicting latent profile membership while preserving the latent profile solution and correcting for classification error.

Then, differences among latent profiles on future-oriented psychological resources and well-being were examined using the Bolck–Croon–Hagenaars (BCH) procedure (Bolck et al., 2004; Asparouhov & Muthén, 2014b). Specifically, latent profiles were compared to future time perspective, meaning in life, and life satisfaction. The BCH method was selected because it accounts for classification uncertainty while providing unbiased estimates of mean differences across profiles on continuous distal outcomes.

## 3. Results

Table 1 reports the means, standard deviations, skewness, kurtosis, and correlations among the study variables. Inspection of skewness and kurtosis values suggested that the variables were approximately normally distributed. Bivariate correlations revealed that future anxiety, climate anxiety, fear of war, and financial threat were positively associated with one another. In contrast, perceived social support, trust in institutions, future time perspective, meaning in life, and life satisfaction were positively correlated and negatively associated with the four threat-related variables.

The LPA was performed using a stepwise procedure. The fit indices of the latent profile models are reported in Table 2. The five-profile solution was retained as the final model. Specifically, the fit indices (AIC, BIC, and SABIC) progressively improved from the two-profile to the five-profile solution. Although the six-profile solution yielded a marginally lower AIC value, both the Lo–Mendell–Rubin adjusted likelihood ratio test (LMR-LRT) and the Bootstrapped Likelihood Ratio Test (BLRT) were no longer significant, indicating that the extraction of an additional profile did not significantly improve model fit. Furthermore, entropy decreased from the five-profile solution (0.913) to the six-profile solution (0.865), suggesting lower classification accuracy.

The retained five-profile solution showed satisfactory fit to the data (AIC = 6457.406; BIC = 6588.575; SABIC = 6499.659), excellent classification accuracy (entropy = 0.913), and significant LMR-LRT and BLRT values. All profiles exceeded the recommended minimum size of 5% of the sample, ranging from 10.9% to 29.6%.

**Figure 1:**
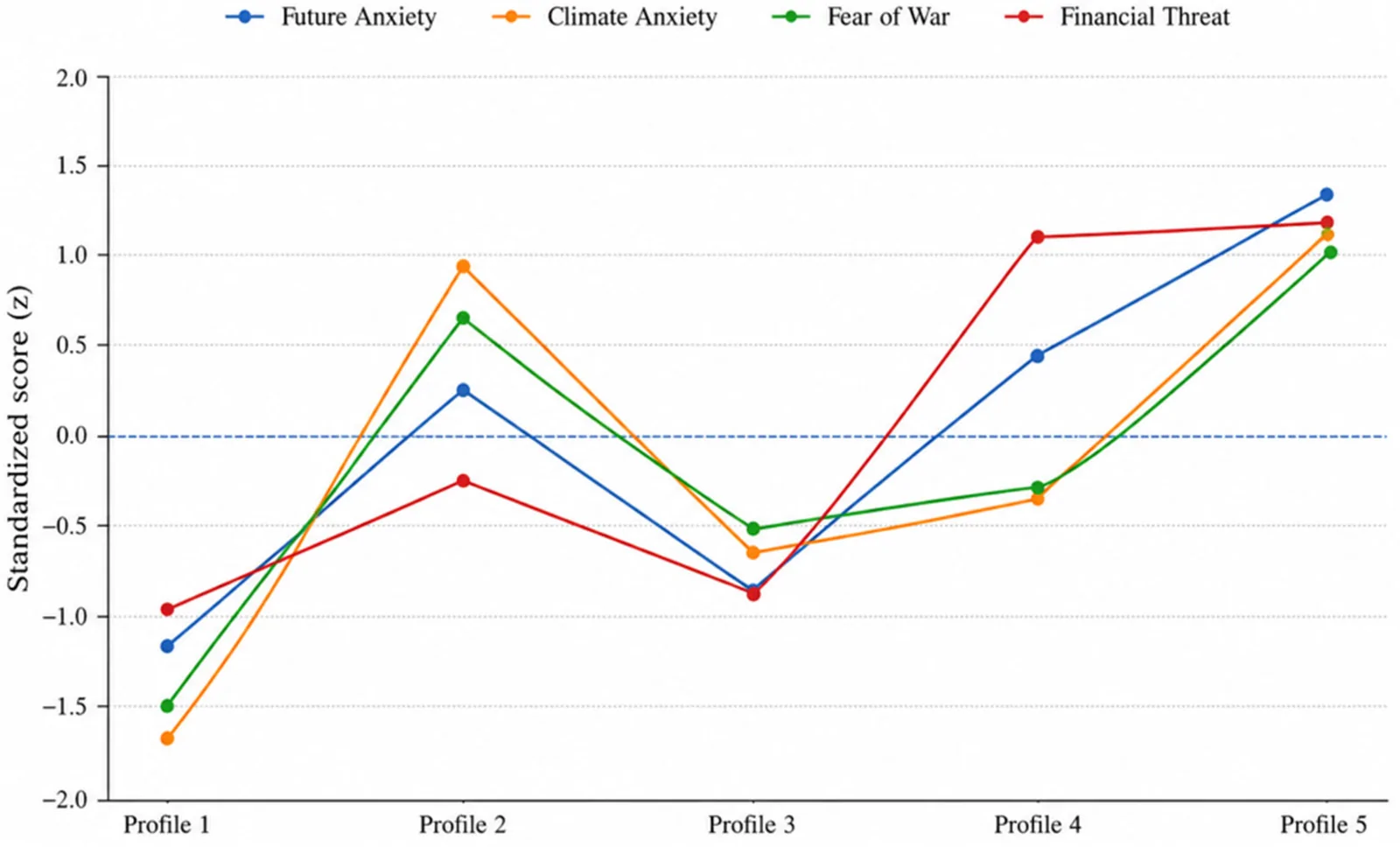
Latent Profiles. **Note**. Values represent standardized scores (z-scores) on the four profile indicators. Profile 1 = Low Threat; Profile 2 = Climate-War Concern; Profile 3 = Moderate Threat; Profile 4 = Financially Focused Threat; Profile 5 = High Threat. The blue dashed horizontal line represents the sample mean (z = 0), indicating the point at which standardized scores equal the overall sample average.

The five-profile solution is displayed in Figure 1. Profile 1 (*n* = 87; 10.9%) was characterized by the lowest levels of future anxiety, climate anxiety, fear of war, and financial threat and was therefore labelled *Low Threat*. Profile 2 (*n* = 199; 24.9%) was characterized by elevated climate anxiety and fear of war, moderately high future anxiety, and comparatively lower levels of financial threat and was labelled *Climate–War Concern*. Profile 3 (*n* = 237; 29.6%) showed relatively low levels of future anxiety and financial threat together with moderate levels of climate anxiety and fear of war and was labelled *Moderate Threat*. Profile 4 (*n* = 159; 19.9%) displayed particularly high levels of financial threat and future anxiety, while climate anxiety and fear of war remained moderate, and was labelled *Financially Focused Threat*. Finally, Profile 5 (*n* = 118; 14.8%) reported the highest levels across all four indicators and was labelled *High Threat*.

To examine whether social resources were associated with latent profile membership, perceived social support and trust in institutions were included as antecedent variables using the R3STEP procedure. The High Threat profile was used as the reference category. Results indicated that both perceived social support and trust in institutions significantly predicted profile membership. Compared with the High Threat profile, students belonging to the Low Threat profile reported significantly higher levels of perceived social support (β = 6.27, SE = 1.17, *p* < 0.001) and trust in institutions (β = 3.52, SE = 0.74, *p* < 0.001). Similarly, membership in the Climate–War Concern profile was associated with higher perceived social support (β = 8.75, SE = 1.20, *p* < 0.001) and trust in institutions (β = 2.98, SE = 0.75, *p* < 0.001) relative to the High Threat profile. Likewise, students classified in the Moderate Threat profile showed significantly higher levels of perceived social support (β = 10.33, SE = 1.23, *p* < 0.001) and trust in institutions (β = 3.96, SE = 0.76, *p* < 0.001) compared with the High Threat profile. Finally, students in the Financially Focused Threat profile also reported significantly higher levels of perceived social support (β = 4.63, SE = 1.09, *p* < 0.001) and trust in institutions (β = 2.11, SE = 0.72, *p* = 0.003) than those in the High Threat profile. Furthermore, additional comparisons among the remaining profiles revealed a more nuanced pattern. Compared with the Low Threat profile, students in the Climate–War Concern profile reported significantly higher levels of perceived social support (β = 2.49, SE = 0.45, *p* < 0.001) but lower levels of trust in institutions (β = −0.54, SE = 0.20, *p* = 0.007). Similarly, students in the Moderate Threat profile reported higher levels of perceived social support than those in the Low Threat profile (β = 4.06, SE = 0.51, *p* < 0.001), whereas differences in trust in institutions were not statistically significant (β = 0.45, SE = 0.23, *p* = 0.052). Additional comparisons showed that students in the Moderate Threat profile reported significantly higher levels of perceived social support (β = 1.57, SE = 0.25, *p* < 0.001) and trust in institutions (β = 0.99, SE = 0.17, *p* < 0.001) than those in the Climate–War Concern profile. Moreover, students in the Financially Focused Threat profile reported significantly lower levels of perceived social support and trust in institutions than those in the Low Threat profile (β = −1.64, SE = 0.40, *p* < 0.001; β = −1.41, SE = 0.22, *p* < 0.001, respectively), the Climate–War Concern profile (β = −4.12, SE = 0.47, *p* < 0.001; β = −0.87, SE = 0.23, *p* < 0.001, respectively), and the Moderate Threat profile (β = −5.70, SE = 0.54, *p* < 0.001; β = −1.86, SE = 0.27, *p* < 0.001, respectively).

Differences among latent profiles on future time perspective, meaning in life, and life satisfaction were examined using the BCH procedure. Significant differences emerged across profiles for all three distal outcomes. For Future Time Perspective, the overall BCH test was significant (χ^2^_(4)_ = 1577.86, *p* < 0.001). Students with a Moderate Threat profile reported the highest scores for future time perspective (M = 3.86, SE = 0.02), followed by Climate–War Concern (M = 3.72, SE = 0.03), Low Threat (M = 3.42, SE = 0.04), and Financially Focused Threat (M = 3.06, SE = 0.03) profiles. High Threat profile students reported the lowest levels (M = 2.30, SE = 0.04). Pairwise comparisons indicated that all profile differences were statistically significant (all *p*s < 0.001). For Meaning in Life, significant differences were also observed across profiles (χ^2^_(4)_ = 805.71, *p* < 0.001). Students with a Moderate Threat profile reported the highest scores for meaning in life (M = 5.03, SE = 0.04), followed by Climate–War Concern (M = 4.70, SE = 0.05), Low Threat (M = 4.43, SE = 0.07), Financially Focused Threat (M = 3.99, SE = 0.04), and High Threat students (M = 3.30, SE = 0.06). All pairwise comparisons were statistically significant, including the comparison between Low Threat and Climate–War Concern, χ^2^ = 9.80, *p* = 0.002. For Life Satisfaction, the overall BCH test was significant, χ^2^_(4)_ = 1117.04, *p* < 0.001. Students with a Moderate Threat profile reported the highest levels of life satisfaction (M = 5.10, SE = 0.04), followed by Climate–War Concern (M = 4.72, SE = 0.04) and Low Threat (M = 4.61, SE = 0.07) profiles. Financially Focused Threat students reported lower levels of life satisfaction (M = 3.88, SE = 0.05), whereas High Threat profile students reported the lowest levels (M = 2.93, SE = 0.06). Pairwise comparisons indicated significant differences among all profiles (*p*s < 0.001), with the exception of the comparison between Low Threat and Climate–War Concern profiles, which was not statistically significant (χ^2^ = 1.97, *p* = 0.160).

## 4. Discussion

The present study adopted a person-centered approach to examine how university students organize perceptions of contemporary societal threats, including climate anxiety, fear of war, financial threat, and future anxiety. The findings contribute to our understanding of the landscape of polycrises in which emerging adults are immersed, highlighting that these phenomena do not affect emerging adulthood as isolated experiences, but rather as interconnected dimensions of a broader experience of uncertainty. Consistent with the theoretical framework guiding this study, which is grounded in the VUCA paradigm (Bennett & Lemoine, 2014), the risk society perspective (Beck, 1992), and the concept of polycrisis (Morin & Kern, 1993; Tooze, 2021, 2022), the identified profiles suggest that contemporary uncertainty is experienced in a multidimensional and co-occurring manner. Rather than reflecting isolated concerns, different forms of social threat appear to cluster within individuals into systematically distinct configurations, supporting the idea that global crises jointly contribute to shaping the future expectations of young adults (e.g., Barchielli et al., 2022; Carta et al., 2022; Kałwak et al., 2024). These findings of distinct profiles characterized by varying levels of perceived social threat further extends the findings of the variable-centered literature, which has consistently associated individual constructs—such as climate anxiety, fear of war, financial threat, and future anxiety—with poorer psychological adjustment and higher levels of distress (e.g., Awad et al., 2024; Clayton & Karazsia, 2020; Hajek et al., 2023; Ogunbode et al., 2022; Regnoli et al., 2024a, 2024b, 2024c; Ryu & Fan, 2023).

Five distinct profiles emerged, indicating that climate anxiety, fear of war, financial threat, and future anxiety combine into qualitatively different patterns rather than reflecting a simple continuum of concern. The Low Threat profile was characterized by consistently low levels across all four indicators, suggesting limited concern about current societal challenges. At the other end of the spectrum, the High Threat profile reported elevated scores across every dimension, reflecting a generalized perception of uncertainty extending across multiple domains. Between these two extremes, three intermediate profiles emerged. The Climate–War Concern profile was characterized by heightened climate anxiety and fear of war but comparatively lower financial threat, whereas the Financially Focused Threat profile showed the opposite pattern, with financial insecurity and future anxiety representing the most salient concerns. Finally, the Moderate Threat profile combined moderate climate- and war-related concerns with relatively low future anxiety and financial threat. Together, these findings suggest that young adults differ not only in the intensity of perceived societal threats but also in the way different concerns are organized within individuals. The most noteworthy finding concerned the Moderate Threat profile. Contrary to the expectation that the most adaptive students would be those reporting the lowest levels of perceived threat, participants in this profile consistently reported the highest scores for future time perspective, meaning in life, life satisfaction, perceived social support, and trust in institutions. These students did not simply disregard contemporary societal challenges. Instead, they reported moderate concern about climate change and geopolitical instability while maintaining relatively low levels of future anxiety and financial threat. This pattern suggests that awareness of collective societal problems is not inherently incompatible with positive psychological functioning. Importantly, the adaptive value of these concerns may depend not only on their intensity but also on their qualitative characteristics. Moderate levels of concern may be less psychologically burdensome than either very low or very high levels of concern, allowing individuals to remain engaged with societal challenges without becoming overwhelmed. Moreover, one possible explanation is that dispositional factors, such as adaptive coping tendencies and personal resources, shape the way societal concerns are experienced and managed (Regnoli et al., 2026a). In this regard, concern about societal threats may function not only as a source of distress but also as a motivational resource that promotes engagement, meaning-making, and future-oriented action among individuals with stronger psychological resources. Rather, adaptive functioning may depend on the ability to acknowledge societal uncertainty without allowing it to undermine confidence in one’s personal future. In other words, the most adaptive students were not those who perceived the world as free of threats, but those who appeared able to integrate those threats into a broader and still positive representation of their future.

This interpretation is broadly consistent with recent person-centered research on climate anxiety, which has repeatedly shown that moderate levels of climate-related concern are often associated with better psychological adjustment than either disengagement or pervasive distress (Chen et al., 2026; Ferrajão et al., 2026; Karademas et al., 2026). Together, these studies suggest that climate concern should not be viewed as uniformly maladaptive, but rather as a response whose psychological implications depend on its intensity and its relationship with other personal resources.

The present findings extend this perspective in two important ways. First, they show that this pattern is not limited to climate anxiety but also emerges when multiple societal threats are considered simultaneously. Second, they suggest that adaptive functioning is shaped less by any single concern than by the overall configuration of concerns within the individual. Rather than indicating that one societal threat is inherently more harmful than another, the present results highlight the importance of considering how different concerns coexist and interact in shaping future-oriented psychological functioning. This broader perspective is particularly relevant in the context of the contemporary polycrisis, where young adults are simultaneously exposed to multiple and overlapping sources of societal uncertainty.

The *High Threat* profile offers an additional and important perspective on young adults’ adaptation to social uncertainty. Students belonging to this group consistently reported the lowest scores for future time perspective, meaning in life, life satisfaction, perceived social support, and institutional trust, suggesting that a generalized perception of societal threats is associated with a substantial reduction in both psychological and contextual resources. This profile appears consistent with previous research that, predominantly using variable-centered approaches, has documented the negative impact of individual societal threats on psychological well-being (e.g., Bonanomi & Rosina, 2022; Clayton & Karazsia, 2020; Hajek et al., 2023; Jannini et al., 2022; Mottola et al., 2023; Mutia & Hargiana, 2021; Regnoli et al., 2024a, 2024b).

Rather than reflecting concern about a single specific domain, this profile may represent a broader cognitive and emotional orientation in which multiple sources of uncertainty converge into a pervasive sense of perceived vulnerability. This interpretation is consistent with the concept of polycrisis (Morin & Kern, 1993; Tooze, 2021, 2022), according to which global challenges are not experienced as isolated events but as interconnected threats that jointly shape individuals’ future expectations. Viewed in this way, more adverse psychological outcomes do not appear to result from exposure to a single societal threat, but rather from the accumulation and integration of multiple concerns into a global representation of uncertainty, in line with recent evidence highlighting the cumulative nature of contemporary crises (Barchielli et al., 2022; Carta et al., 2022; Kałwak et al., 2024).

The comparison between the Climate–War Concern and Financially Focused Threat profiles further illustrates the heterogeneity of young adults’ responses to societal uncertainty. Although both profiles reported elevated levels of perceived threat, they differed markedly in their psychological functioning. Students with the Climate–War Concern profile reported relatively high levels of future-oriented resources and well-being despite elevated concerns about climate change and armed conflict. In contrast, those with the Financially Focused Threat profile consistently reported lower scores for future time perspective, meaning in life, life satisfaction, perceived social support, and trust in institutions.

A possible interpretation is that different societal threats vary in the extent to which they are perceived as directly relevant to personal developmental goals. During emerging adulthood, concerns related to financial security tend to be closely linked to central developmental tasks, such as transitions in education and employment, achieving residential independence, and, more broadly, establishing economic and personal stability (Arnett, 2000; Schulenberg et al., 2004), to the point of eliciting specific concerns within this population (Istituto Toniolo, 2024). By contrast, concerns such as climate change or geopolitical instability, although potentially highly distressing, may be experienced as more distal and collective threats, whose consequences are perceived as less directly connected to immediate personal decisions. This distinction is consistent with construal level theory, which posits that psychological distance influences how events are mentally represented, fostering more abstract representations for events perceived as distant and more concrete representations for events perceived as proximal (Trope & Liberman, 2010). In this respect, this framework may help explain differences across the profiles identified in the present study, particularly regarding the varying weight that different societal threats assume in shaping individual configurations of concern. It is further consistent with the literature on future orientation, which highlights how perceived personal relevance of threats influences motivation, planning, and behavioral regulation (e.g., Zaleski, 1996; Shipp et al., 2009; Fonseca et al., 2020). This interpretation remains speculative and should be examined directly in future research. Nevertheless, it may help explain why profiles characterized by similar overall levels of perceived societal threat differed substantially in future-oriented psychological functioning.

The systematic differences observed across future-oriented psychological resources further clarify the meaning of the identified profiles. Future time perspective, meaning in life, and life satisfaction were not merely correlates of perceived societal threat; rather, they appeared to characterize different ways of relating to the future. Students with the Moderate Threat profile consistently showed the most favorable pattern across all three indicators, whereas those with the High Threat profile showed the least favorable pattern. These findings suggest that future-oriented functioning may represent a key dimension through which societal uncertainty influences psychological adjustment during emerging adulthood.

Importantly, future time perspective and meaning in life are not merely indicators of well-being but psychological resources that enable individuals to maintain long-term goals despite uncertainty (Sica et al., 2024). Young adults who continue to perceive their future as meaningful, coherent, and worth investing in may be better equipped to acknowledge societal challenges without becoming overwhelmed by them (Parola et al., 2022; Sica et al., 2024). Conversely, generalized perceptions of societal threat may compromise the ability to construct positive expectations about the future, thereby reducing both well-being and motivation to engage in future-oriented developmental tasks.

The findings further highlight the importance of contextual resources in shaping young adults’ responses to social uncertainty. Perceived social support and institutional trust consistently differentiated the identified profiles, with the lowest levels observed in the *High Threat* profile and the highest levels in the *Moderate Threat* profile. These results suggest that adaptation to contemporary societal challenges depends not only on how threats are perceived, but also on the broader social context within which such threats are interpreted. Perceived social support may facilitate adaptation by providing emotional regulation, reassurance, and opportunities for shared meaning-making, consistent with the literature on the buffering effect of social support on stress (Cohen & Wills, 1985; Thoits, 2011). Similarly, institutional trust may contribute to the perception that societal challenges are manageable, strengthening the belief that collective actors are able to respond effectively to crises, thereby reducing perceived uncertainty (Newton & Zmerli, 2011; Yang & Bae, 2022). Taken together, these resources may help preserve a future-oriented perspective even in the presence of high levels of perceived societal threat. This interpretation is consistent with ecological perspectives on human development, which emphasize that adaptation emerges from the dynamic interaction between individual and contextual resources rather than from personal characteristics alone (Bronfenbrenner, 1979; Bronfenbrenner & Morris, 2007). In the present study, students showing the most adaptive psychological functioning were also those reporting higher interpersonal and institutional resources, suggesting that trust in both close relationships and broader social systems may protect against the development of generalized perceptions of social threat.

Taken together, the present findings offer a broader perspective on how young adults respond to contemporary societal uncertainty. Rather than supporting the view that societal threats exert uniformly negative psychological effects, the results indicate that these threats are organized into qualitatively different patterns associated with distinct levels of psychological functioning. The most adaptive students were not those who denied or minimized societal challenges, but those who appeared able to acknowledge them while maintaining confidence in their personal future and in the social resources available to cope with uncertainty.

From this perspective, the present study extends existing research by shifting attention from individual societal threats to the broader psychological organization of those threats. A person-centered approach suggests that vulnerability and adaptation are not simply opposite ends of a single continuum of perceived threat. Instead, they reflect different ways of integrating multiple societal concerns into one’s expectations about the future. This distinction is particularly relevant in the context of the contemporary polycrisis, where climate change, geopolitical instability, economic insecurity, and uncertainty about the future rarely occur in isolation but are experienced simultaneously (Barchielli et al., 2022; Carta et al., 2022; Kałwak et al., 2024).

Ultimately, the present findings suggest that the psychological challenge posed by the contemporary polycrisis lies not simply in the accumulation of societal threats, but in the different ways young adults integrate those threats into their representations of the future. Understanding these different pathways may help explain why some young adults remain engaged, hopeful, and future-oriented despite recognizing major societal challenges, whereas others experience the same context as pervasive uncertainty associated with reduced psychological and social resources. This perspective may also inform interventions aimed not only at reducing distress but at strengthening the future-oriented and contextual resources that enable young adults to navigate an increasingly uncertain world.

Several limitations should be considered when interpreting the present findings. First, the cross-sectional design precludes conclusions regarding the temporal ordering of the observed associations. Although the identified profiles differed systematically in psychological and contextual resources, their temporal stability remains to be established. Because the data were collected at a specific historical moment, both profile membership and the relative prevalence of the identified configurations may be partly sensitive to contingent sociopolitical circumstances, including ongoing armed conflicts, economic instability, and climate-related events. On the one hand, this context sensitivity limits the extent to which the profiles can be generalized beyond the period examined; on the other hand, it represents a meaningful feature of the study as the findings provide a contextually grounded psychological snapshot of how young adults organize and experience the multiple sources of uncertainty characterizing the current polycrisis landscape. Longitudinal studies involving repeated assessments across different historical periods are therefore needed to determine whether individuals transition between profiles over time, which configurations remain relatively stable or change in response to broader contextual developments, and whether such transitions are accompanied by changes in future-oriented functioning and well-being. Second, participants were recruited through convenience sampling from universities that agreed to disseminate the study invitation. Consequently, the sample may not be representative of the broader population of Italian university students, limiting the generalizability of the findings. Future research should investigate whether similar profiles emerge in more diverse educational, occupational, and cultural contexts, as well as across different stages of the life span. Finally, the present study focused on four major societal threats that are particularly salient in the current sociohistorical context. Expanding this framework to include additional sources of uncertainty—such as housing insecurity, technological change, political polarization, or public health concerns—may provide a more comprehensive understanding of how contemporary societal challenges are psychologically organized. An additional limitation concerns the use of two measures—the Financial Threat Scale and the Trust in Institutions measure—which had not previously been validated in the Italian context. However, to assess their adequacy in the present sample, their factorial structure was examined through confirmatory factor analyses, which yielded good-to-excellent fit indices. These findings provide preliminary support for the psychometric robustness of the measures and for their suitability within the context of the present study. Nevertheless, a broader validation in the Italian context would further consolidate their psychometric properties and strengthen the generalizability of the findings. Future research could therefore examine this issue in greater depth and assess the replicability of the identified profile solution in additional Italian samples. Moreover, although the present study adopted a person-centered approach, the findings would benefit from being complemented by a traditional variable-centered perspective. In particular, future research could examine whether psychological and contextual resources (e.g., perceived social support, trust in institutions, future time perspective, meaning in life, and life satisfaction) operate as mediators or moderators in the relationship between societal threats and indicators of psychological adjustment. Such an approach would allow for a more integrated understanding of both the structural configuration and the functional mechanisms underlying the association between societal concerns and well-being in emerging adulthood.

## Figures and Tables

**Table 1:** Descriptive statistics and correlations between study variables.

Variable	M	SD	Skew	Kurt	1	2	3	4	5	6	7	8
**1. Future Anxiety**	2.97	0.96	−0.01	−0.83	-							
**2. Climate Anxiety**	3.28	0.80	−0.16	−0.83	0.61	-						
**3. Fear of war**	3.16	0.84	−0.12	−0.60	0.56	0.72	-					
**4. Financial Threat**	3.01	1.09	0.20	−1.17	0.66	0.39	0.39	-				
**5. Future Time Perspective**	3.38	0.62	−0.77	−0.50	−0.57	−0.29	−0.32	−0.66	-			
**6. Meaning in Life**	4.41	0.80	−0.24	−0.43	−0.50	−0.26	−0.27	−0.59	0.69	-		
**7. Social Support**	4.71	0.64	−0.39	−0.42	−0.56	−0.24	−0.26	−0.65	0.76	0.66	-	
**8. Trust in Institutions**	3.11	0.71	−0.16	−0.17	−0.52	−0.34	−0.34	−0.54	0.53	0.45	0.54	-
**9. Life Satisfaction**	4.38	0.92	−0.52	−0.16	−0.60	−0.31	−0.34	−0.64	0.73	0.63	0.69	0.52

**Note**. All correlations were statistically significant (*p* < 0.001). M = mean; SD = standard deviation; Skew = skewness; Kurt = kurtosis.

**Table 2:** Indices of latent profile solutions.

Profile	AIC	BIC	SABIC	Entropy	LMR-LRT *p*	BLRT *p*	Smallest Class (%)
**2**	7346.996	7407.896	7366.614	0.906	<0.001	<0.001	40.9
**3**	7018.914	7103.237	7046.076	0.903	<0.001	<0.001	24.9
**4**	6653.282	6761.028	6687.990	0.923	<0.001	<0.001	19.9
**5**	**6457.406**	**6588.575**	**6499.659**	**0.913**	**<0.001**	**<0.001**	**10.9**
**6**	6454.609	6609.201	6504.408	0.865	0.500	0.200	5.3

**Note**. AIC = Akaike Information Criterion; BIC = Bayesian Information Criterion; SABIC = Sample-Size Adjusted Bayesian Information Criterion; LMR-LRT = Lo–Mendell–Rubin Adjusted Likelihood Ratio Test; BLRT = Bootstrapped Likelihood Ratio Test.

## Data Availability

The data that support the findings of this study are available from the corresponding author upon reasonable request.
